# Influence of stress induced by the first announced state of emergency due to coronavirus disease 2019 on outpatient blood pressure management in Japan

**DOI:** 10.1038/s41440-021-00832-w

**Published:** 2021-12-24

**Authors:** Kazuo Kobayashi, Keiichi Chin, Shinichi Umezawa, Shun Ito, Hareaki Yamamoto, Shiro Nakano, Nobukazu Takada, Nobuo Hatori, Kouichi Tamura

**Affiliations:** 1 Sagamihara Physicians Association, Sagamihara, Japan; 2 Department of Medical Science and Cardiorenal Medicine, Yokohama City University Graduate School of Medicine, Kanagawa, Japan; 3 Kobayashi hospital, Odawara, Japan

**Keywords:** Coronavirus disease-2019, A state of emergency, White coat hypertension

## Abstract

To prevent further spread of coronavirus disease 2019 (COVID-19), the Japanese government announced a state of emergency, resulting in major stress for the population. The aim of this study was to investigate a possible association between changes in daily stress and blood pressure (BP) in Japanese patients. We retrospectively investigated 748 patients with chronic disease who were treated by the Sagamihara Physicians Association to determine changes in stress during the COVID-19 state of emergency from 7 April to 31 May 2020. During the state of emergency, office BP significantly increased from 136.5 ± 17.5/78.2 ± 12.0 to 138.6 ± 18.6/79.0 ± 12.2 (*p* < 0.001 and *p* = 0.03, respectively). In contrast, home BP significantly decreased from 128.2 ± 10.3/75.8 ± 8.8 to 126.9 ± 10.2/75.2 ± 9.0 (*p* < 0.001 and *p* = 0.01, respectively), and the ratio of white coat hypertension was significantly increased (*p* < 0.001). Fifty-eight percent of patients worried about adverse effects of hypertension as a condition contributing to the severity and poor prognosis of COVID-19; decreased amounts of exercise and worsened diet compositions were observed in 39% and 17% of patients, respectively. In conclusion, a significant increase in office BP with the white coat phenomenon was observed during the state of emergency, as well as an increase in related stress. To prevent cardiovascular events, general practitioners should pay more attention to BP management during stressful global events, including the COVID-19 pandemic.

## Introduction

Severe acute respiratory syndrome coronavirus 2 (SARS-CoV-2) caused a global coronavirus disease-2019 (COVID-19) pandemic, as declared by the World Health Organization on March 11, 2020. COVID-19 was confirmed for the first time in January 2020, and despite epidemic prevention efforts, the number of patients with COVID-19 continued to increase, primarily in metropolitan areas. To prevent the further spread of COVID-19, the Japanese government announced a state of emergency on 7 April 2020, similar to the “lockdowns” implemented in other countries. This state of emergency strongly encouraged to stay home or maintain social distancing to reduce person-to-person contact during daily societal activities. Accordingly, an increase in daily stress was expected to occur, especially in chronically ill patients who were already experiencing daily stress due to their illness. Natural disasters, such as earthquakes or typhoons, often affect Japan, and previous studies have suggested that these events increase stress in Japanese patients. For example, Kario et al. reported that white coat hypertension changed to persistent hypertension in three patients immediately following the Hanshin–Awaji earthquake in 1995 [1]. They also reported a significant increase in heart disease events following this disaster [2]. Miyakawa et al. reported that home blood pressure (BP) worsened in patients who lived within a certain distance from the disaster area during the East Japan Earthquake in 2011 [3]. This evidence gave rise to the concept of disaster-related diseases, such as stress-related cardiomyopathy (Takotsubo cardiomyopathy, sudden death, pulmonary embolism), in victims of disasters. Furthermore, it is considered necessary to prioritize the care of hypertension-related diseases (stroke, ischemic coronary artery disease, heart failure, or aortic dissection) not only immediately after disasters but also during reconstruction [4]. Although the COVID-19 situation is different from conventional natural disasters such as earthquakes or typhoons, there is concern that the state of emergency declaration may cause increases in various stresses experienced by patients. As a result, patients may need more attention than they usually require from general practitioners. Hypertension is one of the most common diseases; the prevalence of hypertension among men and women aged 40–74 years is 60% in males and 41% in females [5], and the number of patients with hypertension is estimated to be 43 million in Japan. It is necessary for general practitioners to properly understand the influence of stress caused by the state of emergency on BP management. However, there is little evidence concerning the influence of the COVID-19 pandemic on BP management, especially during the state of emergency.

In this study, we hypothesized that a state of emergency induces stress in patients, changes their BP profile, and leads to health hazards. It is important for general practitioners to understand how BP profiles change in unusual circumstances. Furthermore, it is necessary to discuss strategies for BP control under unusual circumstances to prevent organ damage. The aim of this study was to evaluate the stress of patients during the COVID-19 state of emergency and to clarify its influence on BP.

## Methods

### Study population

The study included 919 outpatients who visited member facilities of the Sagamihara Physicians Association during the first COVID-19 state of emergency (from 7 April to 31 May 2020). The inclusion criteria were as follows: (1) patients who regularly visited clinics for any lifestyle-related or chronic diseases; (2) patients aged over 20 years; (3) patients with blood pressure (BP) data measured in office prior to the state of emergency announcement (data from January to March 2020) and data available at the time of the survey; and (4) patients who answered a questionnaire related to daily stress levels during the state of emergency. The exclusion criteria were (1) patients who visited the clinic for any acute or emergency disease or (2) patients who expressed an intent to opt-out during the study. On the basis of the above criteria, 171 patients were excluded from the study (51 patients did not have sufficient data during the study period, 7 patients lacked BP data, and 116 patients did not completely answer the questionnaire). A total of 748 patients were included in the present analysis. Among the 748 patients, 535 patients had early morning BP measurements recorded at home. Out of 546 patients, 535 patients had early morning BP data measured during the period from January to March 2020. BP data from April to June 2019 were not necessarily needed; however, comparisons between BP measurements from the last year (from April to June 2019), during the latest visit (from January to March 2020), and during the state of emergency were performed with 567 patients for office BP and 347 patients for home BP.

### BP measurements and the clinical data

Office BP measurements were performed at each institution at one visit during the state of emergency using validated cuff oscillometric devices. According to the Japanese Society of Hypertension guidelines for the management of hypertension (JSH 2019) [6], office BP measurements were performed in a quiet environment after allowing the patient to rest for a few minutes in a seated position with uncrossed legs. The average of two consecutive measurements taken 1–2 min apart was defined as the office BP. Home BP measurements were also performed according to the JSH 2019 guidelines [6] using oscillometric devices with upper arm cuffs. The patients performed home BP measurements every morning, and the average values of the home BP measurements during the week before the clinic visit were calculated. According to the 2019 JSH guidelines [6], to analyze BP distribution depending on a patient’s target BP, the patients were divided into four groups based on the target BP measured both in the office and at home. Among the patients with diabetes mellitus (DM), cerebro- or cardiovascular disease, chronic kidney disease with proteinuria, or patients under 75 years old, each group was identified as follows: the controlled hypertension group (office BP < 130/80 and home BP < 125/75 mmHg), masked hypertension group (office BP < 130/80 and home BP ≥ 125/75 mmHg), white coat hypertension group (office BP ≥ 130/80 and home BP < 125/75 mmHg), and sustained hypertension group (office BP ≥ 130/80 and home BP ≥ 125/75 mmHg). Among the remaining patients who were not mentioned above, 10 mmHg was added to the above BP thresholds in the office and at home. These thresholds were considered the target BP level.

To evaluate changes in BP, the following parameters were recorded both before and during the state of emergency: age, sex, body weight (BW), serum creatinine level, glycated hemoglobin A_1c_ (HbA_1c_) level, urinary protein level (urine albumin-to-creatinine (ACR) or qualitative proteinuria), N-terminal pro-brain natriuretic peptide (NT-proBNP), and estimated salt intake. The estimated glomerular filtration rate (eGFR) was calculated using the following formula: eGFR (mL/min/1.73 m^2^) = 194 × age^−0.287^ × serum creatinine^−1.094^ × (0.739 for women) [7].

In this study, the qualitative proteinuria values were converted to albuminuria values using the formula reported by Sumida et al. [8]. Estimated salt intake was calculated using Tanaka’s formula using spot urine [9].

### Questionnaire related to changes in stress following the announcement of the COVID-19 state of emergency

There are no previous reports on what kind of stress should be evaluated under the state of emergency associated with the COVID-19 pandemic. Based on the detailed analysis of the Great Hanshin–Awaji Earthquake, Kario et al. reviewed the relationship between earthquake disasters and cardiovascular diseases [4, 10]. Furthermore, they developed the Disaster Cardiovascular Prevention (DCAP) network, which was initially established to prevent cardiovascular death due to the Great East Japan Earthquake [11]. The cardiovascular prevention score (SEDWITHP8) was proposed, which contained eight questions worth one point each; a score of more than six points recommends the prevention of cardiovascular disease [4, 12]. Because the current state of emergency is not the same as disasters such as earthquakes, we conducted a survey with reference to the management of disaster-associated cardiovascular prevention score [12]. The survey contained nine questions that were related to stress and were suspected to be affected by the COVID-19 pandemic or the state of emergency announcement. The question topics were as follows: fear of the relationship between COVID-19 and hypertension, daily stress, diet, salt intake, frequency of lunch or dinner eaten at home, amount of exercise, amount of alcohol intake, quality of sleep, and adherence to taking medications. Due to the limitations under the state of emergency, we performed the questionnaire so that patients could easily select the answers on a five-point scale: much worse, a little worse, no change, a little improved, and much improved. Moreover, the distribution of these questionnaires aimed to give advice or medications to patients who answered “worse” despite the limited consultation time. Furthermore, we analyzed the total scores of these nine factors to assess stress and related changes in BP. The answers to the nine questions were changed to discrete variables, scored from −2 to +2 points, and summed to determine the total stress score. We distributed this questionnaire as a part of our regular clinical practice for patients to control their health conditions and BP status under the state of emergency. Therefore, informed consent was not needed for the distribution of questionnaires.

### Statistical analysis

IBM SPSS Statistics software (version 25.0; IBM Inc., Armonk, NY) was used for statistical analysis. Data that showed normal distributions are reported as the mean ± standard deviation (SD), while those that showed skewed distributions are reported as the median (lower quartile, upper quartile). Differences between two time points were compared using a paired sample t test for parametric data, a Wilcoxon signed-rank test for nonparametric data, and McNemar’s test for the nominal value. A *p* value less than 0.05 was considered significant. Differences among three serial points of data that consisted of continuous values were tested by repeated measures analysis of variance (ANOVA), and differences among three serial points of data that consisted of nominal values were tested by the Cochran Q test. If the results were significant (*p* < 0.05), further analysis was performed as a post hoc test with the Bonferroni correction. A chi-square test was performed to compare the BP distribution depending on the patient’s target BP divided into four groups (controlled, masked hypertension, white coat hypertension, and sustained hypertension) between January and May 2020 and under the state of emergency.

### Multivariable linear regression analysis of change in mean arterial pressure (MAP)

Multivariable linear regression analysis was performed to identify the correlates of the change in office MAP or home MAP with potential predictors. MAP values, which involve both systolic BP and diastolic BP, were used in this study and were calculated as “(systolic BP − diastolic BP)/3 + diastolic BP”. Among the data that were collected in this survey, we selected the factors that were thought to be related to the change in BP as covariates in the multivariable linear regression analysis. These included concomitant medications and various clinical parameters, such as age, sex, BW before the state of emergency announcement, MAP from January to March 2020, the eGFR, estimated salt intake, DM status, use of antihypertensive drugs, and the answers to the nine questions that were changed to discrete variables.

### Receiver operating characteristic (ROC) analysis for calculating the total stress score cutoff value

We assessed the relationship between the change in MAP during the state of emergency and the total stress score. Receiver operating characteristic (ROC) analysis was used to examine the overall predictive accuracy of the increase in MAP and the total stress score. The results are reported as the area under the curve (AUC). The cutoff value of the total stress score selected for further analysis was determined from the results of the ROC analysis.

### Binary classification and characterization based on the total stress score

We divided the patients into two groups on the basis of the total stress score. The patients with total stress scores higher than the cutoff value determined during the ROC analysis were compared with the patients with total stress scores lower than the cutoff value. Analysis of covariance (ANCOVA) was performed to compare the clinical parameters between the two groups. Clinical parameters measured before the state of emergency announcement were treated as covariates, those measured during the state of emergency were treated as dependent variables, and the groups were divided by the total stress score.

### Ethics statement

This study was conducted in compliance with the Declaration of Helsinki, and the Special Ethics Committee of the Kanagawa Medical Association, Japan approved this study (Japan Physicians Association of Internal Medicine 028-2008-001, December 4, 2020).

## Results

### Patient demographics

The clinical background data of the patients assessed during the state of emergency are shown in Table 1. The mean age was 67.3 ± 12.3 years (range from 22 to 97 years), with 424 males and 324 females. Of these patients, 714 patients (96%) were hypertensive, and 423 patients (57%) were diabetic.

**Table 1:** Clinical background of cases during the state of emergency due to COVID-19.

Total (*n* = 748)	Analyzed cases	During the state of emergency
Male (%)	748	424 (57%)
Age (years-old)	748	67.3 ± 12.3 (range, 22–97)
Current smoke	520	31 (6%)
Hypertensin	748	714 (95%)
Diabetes mellitus	748	423 (57%)
Dyslipidemia	610	544 (89%)
Chronic kidney disease	610	336 (55%)
Cardiovascular disease	610	104 (17%)
Cerebrovascular disease	610	40 (7%)
BMI	519	24.0 ± 3.5
BW (kg)	715	63.9 ± 12.8
Office SBP/DBP (mmHg)	748	138.6 ± 18.6/ 79.0 ± 12.2
Office MAP (mmHg)	748	99.0 ± 12.5
Pulse rate	520	79.2 ± 13.2
Home SBP/DBP (mmHg)	546	126.8 ± 10.3/ 75.3 ± 9.1
Home MAP (mmHg)	546	92.5 ± 8.2
eGFR (mL/min/1.73 m^2^)	565	66.9 ± 17.8
HbA_1c_(mmol/mol (%))	546	48.1 ± 10.1 (6.5 ± 0.9)
Urine albumin-to creatinine ratio (mg/gCr)	391	9.5 [5.4, 26.1]
Urine protein (g/gCr)	79	0.16 [0.05, 0.39]
Estimated Sodium intake (g/day)	503	8.8 [7.2, 10.4]
NT proBNP	178	91.0 [40.5, 193.0]
**Current medications were analyzed in 610 cases**		
Antihypertensive agents		
ARB	308 (51%)	
ACEI	40 (7%)	
Ca channel blocker	297 (49%)	
β blocker	84 (14%)	
Thiazide	22 (4%)	
Mineralocorticoid receptor blocker	45 (7%)	
Loop diuretic	9 (2%)	
hypoglycemic agents		
DPP4 inhibitor	150 (25%)	
Metformin	97 (16%)	
SGLT2 inhibitor	68 (11%)	
Insulin	51 (8%)	
Sulphonyl urea	64 (11%)	
GLP1 receptor agonist	20 (3%)	
Pioglitazone	34 (6%)	
`αGlucosidase inhibitor	70 (12%)	
Glinide	32 (5%)	
Others		
Statin	439 (72%)	
Ezetimibe	92 (15%)	
Fibrate	12 (2%)	
Antiplatelet	126 (21%)	
EPA	34 (6%)	
Anticoagulant	22 (4%)	

Data shown are mean ± standard deviation, median [lower quartile, upper quartile], or numbers (%).

*ACEI* angiotensin converting enzyme inhibitor, *ARB* angiotensin receptor blocker, *COVID-19* corona virus disease 2019, *BMI* body mass index, *BW* body weight, *DBP* diastolic blood pressure, *DPP4* dipeptidyl peptidase-4, *EPA* eicosapentaenoic acid, *eGFR* estimated glomerular filtration rate, *GLP1* glucagon-like peptide 1, *HbA*_*1c*_ hemoglobin A_1c_, *MAP* mean arterial pressure, *NT proBNP* N-terminal pro-brain natriuretic peptide, *SBP* systolic blood pressure, *SGLT2* sodium-glucose co-transporter 2.

### Comparison of office BP and home BP

Table 2a shows that office systolic BP (SBP) and diastolic BP (DBP) measurements were significantly higher during the state of emergency than the measurements from January to March 2020 (*p* < 0.001 and 0.03, respectively), whereas home SBP and DBP measurements were significantly lower during the state of emergency than the measurements from January to March 2020 (*p* < 0.001 and 0.01, respectively). The achievement rate of target home BP significantly increased during the state of emergency (*p* < 0.001), and the BP distribution was significantly different before and after the state of emergency (*p* < 0.001 by chi-square test). Then, we performed McNemar’s test to compare the prevalence of each BP distribution. Only the prevalence of white coat hypertension patients was significantly higher during the state of emergency than from January to March 2020 (*p* < 0.001; other *p* values were 0.11 in sustained hypertension patients, 0.12 in masked hypertension patients, and 0.57 in controlled hypertension patients).

**Table 2:** The comparison of office BP and home BP. a) Comparison between from January to March 2020 and during the state of emergency in 748 patients. b) Comparison between from April to June 2019, from January to March 2020, and during the state of emergency in 567 patients.

	Jan–Mar in 2020	During the state of emergency	*p* value by paired *t*-test
a) BP			
In office (*n* = 748)			
SBP (mmHg)	136.5 ± 17.5	138.6 ± 18.6	<0.001^a^
DBP (mmHg)	78.2 ± 12.0	79.0 ± 12.2	0.03^a^
MAP (mmHg)	97.6 ± 12.0	98.9 ± 12.5	0.002
The achievement rate of target BP (*n* = 712)	207 (29%)	202 (28%)	0.77^b^
At home (*n* = 535)			
SBP (mmHg)	128.2 ± 10.3	126.9 ± 10.2	<0.001^a^
DBP (mmHg)	75.8 ± 8.8	75.2 ± 9.0	0.01^a^
MAP (mmHg)	93.3 ± 7.9	92.5 ± 8.1	0.001
The achievement rate of target BP (*n* = 516)	123 (24%)	160 (31%)	<0.001^b^
b) BP distribution			
Controlled	71 (13%)	72 (13%)	<0.001^c^
White-coat	70 (13%)	91 (17%)	
Masked	83 (16%)	67 (13%)	
Sustained	313 (58%)	307 (57%)	

	Apr–Jun in 2019	Jan–Mar in 2020	During the state of emergency	*p* value	*p* value by post-hoc analysis with Bonferroni method
In office (*n* = 567)					
SBP (mmHg)	136.2 ± 16.9	137.5 ± 17.7	140.3 ± 18.6	<0.001^d^	<0.001^e^, <0.001^f^
DBP (mmHg)	77.6 ± 11.9	78.3 ± 11.9	76.2 ± 12.4	<0.001^d^	0.007^e^
MAP (mmHg)	99.0 ± 11.2	99.5 ± 11.3	101.4 ± 11.9	<0.001^d^	<0.001^e^, 0.002^f^
The achievement rate of target BP	182 (32%)	163 (29%)	145 (26%)	0.008^g^	0.006^e^
At home (*n* = 347)					
SBP (mmHg)	129.0 ± 8.8	128.3 ± 9.2	127.0 ± 9.2	<0.001^d^	<0.001^e^, <0.001^f^
DBP (mmHg)	75.7 ± 9.2	75.8 ± 7.9	75.1 ± 8.3	<0.001^d^	0.005^f^
MAP (mmHg)	93.5 ± 7.8	93.3 ± 6.9	92.5 ± 7.2	<0.001^d^	0.008^e^, <0.001^f^
The achievement rate of target BP	64 (18%)	81 (23%)	109 (31%)	<0.001^g^	<0.001^e^, 0.001^f^

Data shown are mean ± standard deviation, or numbers (%).

Among patients with diabetes mellitus, cerebro- or cardio-vascular disease, chronic kidney disease with proteinuria, or under 75 years old, each group were identified as controlled hypertension group, office BP < 130/80 and at home BP < 125/75 mmHg; masked hypertension group, office BP < 130/80 and home BP ≥ 125/75 mmHg; white coat hypertension group, office BP ≧ 130/80 and home BP < 125/75 mmHg; and sustained hypertension group, office BP ≥ 130/80 and home BP ≥ 125/75 mmHg. Among the rest patients other than described above, 10 mmHg was added to the BP thresholds in office and at home. These thresholds were considered as the level of the target BP.

*ANOVA* analysis of variance, *DBP* diastolic blood pressure, *MAP* mean arterial pressure, *SBP* systolic blood pressure.

^a^Paired *t*-test.

^b^McNemar’s test.

^c^Chi-square test in Table 2a.

^d^One-way repeated ANOVA.

^e^Comparison between from April to June 2019 and during the state of emergency

^f^Comparison between from January to March 2020 and during the state of emergency

^g^Cochran Q test in Table 2b.

Table 2b shows the results of ANOVA comparing the BP measurements taken from April to June 2019, from January to March 2020, and during the state of emergency. A significant increase in office SBP measurements and a significant decrease in home SBP measurements during the state of emergency were observed compared to measurements from January to March 2020 and from April to June 2019 (*p* < 0.001 in all cases).

Table 3 shows the comparison of other clinical findings before and after the announcement of the state of emergency. A significant decrease in eGFR and a significant increase in NT-proBNP were observed during and after the state of emergency (*p* = 0.003 and 0.01, respectively). Table 4 shows the patients’ answers to the questions related to the change in stress after the state of emergency. Fifty-eight percent of the patients worried about adverse effects of hypertension as a condition contributing to the severity and poor prognosis of COVID-19, and decreased amounts of exercise and worsened diet compositions were observed in 39% and 17% of the patients, respectively.

**Table 3:** Comparisons of other clinical findings before and after the state of emergency.

	Analyzed cases	Before the state of emergency	During or after the state of emergency	*p* value by paired *t*-test
BW (kg)	690	64.1 ± 12.7	64.0 ± 12.8	0.09
Pulse rate (counts/min)	520	79.1 ± 13.2	79.2 ± 13.2	0.80
eGFR (mL/min/m^2^)	584	68.3 ± 18.1	67.3 ± 18.2	0.003
HbA_1c_ (mmol/mol(%))	536	47.9 ± 10.5 (6.5 ± 1.0)	48.2 ± 10.1 (6.6 ± 0.9)	0.12
Logarithmic value of urine albumin to creatinine ratio (mg/gCr)	393	2.56 ± 1.35	2.59 ± 1.32	0.40
Urine protein (g/gCr)	72	0.37 ± 0.66	0.38 ± 0.86	0.87
Logarithmic value of NT-proBNP	175	4.58 ± 1.13	4.69 ± 1.14	0.01
Estimated salt intake (g/day)	469	9.1 ± 2.0	8.9 ± 2.5	0.09

Data shown are mean ± standard deviation.

*BW* body weight, *eGFR* estimated glomerular filtration rate, *HbA*_*1c*_ hemoglobin A_1c_, *NT-proBNP* N-terminal pro-brain natriuretic peptide.

**Table 4:** Questions related to the change in stress after the state of emergency

Q1. Do you worry about adverse effects of hypertension as a contributing condition to the severity and poor prognosis of COVID-19?						
	No, never	Yes, a little	neither yes or no	Yes, moderately	Yes, strongly	No answer
	23 (3%)	214 (29%)	37 (5%)	253 (34%)	182 (24%)	39 (5%)
Q2. How did the stress in your daily life change after the declaration of emergency due to the rapid spread of COVID-19?						
	Much decreased	A little decreased	Usual	A little increased	Much increased	No answer
	13 (2%)	28 (4%)	313 (42%)	298 (40%)	87 (11%)	9 (1%)
Q3. How did your lifestyle change after the declaration of emergency due to the rapid spread of COVID-19?						
	Much improved	A little improved	Usual	A little worsened	Much worsened	No answer
Dietary intake	21 (3%)	68 (10%)	533 (70%)	119 (16%)	5 (1%)	2 (0%)
Salt intake	18 (3%)	82 (11%)	599 (80%)	45 (6%)	1 (0%)	3 (0%)
The frequency of dinner or lunch at home	81 (11%)	92 (12%)	554 (74%)	15 (2%)	2 (0%)	4 (1%)
Amount of exercise	26 (4%)	69 (9%)	361 (48%)	209 (28%)	80 (11%)	3 (0%)
Quality of sleep	20 (3%)	49 (7%)	572 (76%)	94 (13%)	9 (1%)	4 (0%)
Amount of alcohol intake	59 (8%)	46 (6%)	597 (80%)	28 (4%)	4 (0%)	14 (2%)
Adherence to taking medications	17 (2%)	12 (2%)	690 (92%)	19 (3%)	2 (0%)	8 (1%)
The stress score for each answer	−2	−1	0	+1	+2	0

*COVID-19* corona virus disease 2019.

### Multiple linear regression analysis for the change in office MAP and home MAP

Multiple linear regression analysis of the change in office MAP measurements identified the following independent factors: office MAP measurements from January to March 2020, mineral corticoid receptor agonist use, worsened dietary intake, and worsened adherence to taking medications, with coefficient values (95% confidence intervals, [CIs]) of −0.42 [−0.50, −0.35], 4.30 [0.95, 7.65], 1.68 [0.25, 3.10], and 2.48 [0.15, 4.81], respectively (*p* value were <0.001, 0.01, 0.02, and 0.04, respectively).. Additionally, multiple linear regression analysis of the change in home MAP identified the following independent factors: home MAP measurements from January to March 2020, BW before the state of emergency announcement, and worsened quality of sleep, with coefficient values (95% CIs) of −0.11 [−0.16, −0.06], 0.05 [0.02, 0.09], 0.82 [0.15, 1.50], and 0.20 [0.003, 0.40], respectively (*p* < 0.001, 0.002, 0.02 and 0.047, respectively).

### Comparisons between the two total stress score groups

Supplementary Fig. S1 shows the distribution of the total stress score, with a median of 1.0 (lower quartile, upper quartile; −1.0, 2.0), of the 674 patients who completed the stress level questionnaire. From the ROC analysis, the cutoff value of the total stress score for an increase in office MAP was 3.0, with a sensitivity of 27.3% and a specificity of 78.2% (Supplementary Fig. S2).

The proportion of males was higher in the total stress score <3 group (*n* = 508) than in the stress score ≥3 group (*n* = 166) (63% and 44%, respectively, *p* < 0.001); however, there was no significant difference in the clinical findings between the two groups (Table 5). From the ANCOVA results, office SBP, office MAP, home DBP, and home MAP were significantly higher in the total stress score ≥3 group than in the total stress score <3 group (*p* = 0.04, 0.048, 0.01, and 0.02, respectively) (Table 5).

**Table 5:** Comparisons between two groups divided by 3 of the total stress score before and after the state of emergency.

	Jun–Mar 2020			During the state of emergency		
The total stress score	<3	≧3		<3	≧3	
	*n* = 508	*n* = 166	*p* value by unpaired *t* test	*n* = 508	*n* = 166	*p* value by ANCOVA
Age	66.7 ± 12.5	67.3 ± 12.1	0.59			
Gender (Male)	318 (63%)	73 (44%)	<0.001			
Office SBP (mmHg)	137.5 ± 18.1	135.3 ± 16.1	0.16	138.7 ± 18.5	140.1 ± 19.4	0.04
Office DBP (mmHg)	78.4 ± 12.2	78.6 ± 11.7	0.88	79.1 ± 11.8	80.4 ± 13.1	0.13
Office MAP (mmHg)	98.1 ± 12.3	97.5 ± 11.4	0.57	99.9 ± 12.3	100.3 ± 13.4	0.048
Home SBP (mmHg)	127.9 ± 10.1	128.6 ± 11.0	0.48	126.3 ± 9.9	127.6 ± 11.3	0.18
Home DBP (mmHg)	76.1 ± 9.1	75.9 ± 8.7	0.85	75.1 ± 9.1	76.2 ± 9.6	0.01
Home MAP (mmHg)	93.3 ± 8.2	93.5 ± 7.9	0.88	92.2 ± 8.0	93.3 ± 9.1	0.02
BW (kg)	64.5 ± 12.4	64.3 ± 14.1	0.87	64.4 ± 12.5	64.4 ± 14.1	0.70
eGFR (mL/min/1.73 m^2^)	69.2 ± 18.0	66.1 ± 17.3	0.08	67.8 ± 18.0	65.7 ± 17.6	0.68
HbA_1c_(mmol/mol (%))	47.7 ± 10.8 (6.5 ± 1.0)	47.6 ± 10.3 (6.5 ± 0.9)	0.92	48.0 ± 10.2 (6.5 ± 0.9)	47.9 ± 9.9 (6.5 ± 0.9)	0.89
The logarithmic value of ACR	2.46 ± 1.36	2.45 ± 1.32	0.95	2.55 ± 1.28	2.65 ± 1.34	0.12
Estimated salt intake (g/day)	9.1 ± 2.1	9.0 ± 2.0	0.70	8.9 ± 2.4	8.8 ± 2.5	0.69
Urine Na/K ratio	1.62 ± 0.86	1.56 ± 0.80	0.55	1.57 ± 1.11	1.63 ± 1.17	0.68

The total stress score	<3	≧3	
	*n* = 508	*n* = 166	*p* value by unpaired *t* test
The total stress score	−0.5 ± 2.1	4.5 ± 1.5	<0.001
The frequency of the answers “much worsening” or “ a little worsening”			*p* value by chi-square test
Daily stress	210 (41%)	145 (87%)	<0.001
Dietary intake	44 (9%)	73 (44%)	<0.001
Salt intake	4 (1%)	38 (23%)	<0.001
Frequency of dinner or lunch at home	7 (1%)	9 (5%)	<0.001
Amount of exercise	143 (29%)	123 (74%)	<0.001
Quality of sleep	39 (8%)	50 (30%)	<0.001
Amount of alcohol intake	12 (2%)	16 (10%)	<0.001
Adherence to taking medications	12 (2%)	5 (3%)	0.64
Feel fear for the relationship between COVID-19 and hypertension	259 (51%)^a^	157 (95%)^a^	<0.001

Data shown are mean ± standard deviation, or numbers (%).

*ANCOVA* analysis of covariance, *ACR* urine albumin-to-creatinine ratio, *BW* body weight, *COVID-19* coronavirus disease, *DBP* diastolic blood pressure, *eGFR* estimated glomerular filtration rate, *HbA1c* hemoglobin A1c, *MAP* mean arterial pressure.

^a^Answers “Yes, moderately” or “Yes, strongly”.

To show the relationship between the change in BP and the total stress score, we also performed a comparison between the quintiles that were defined using the total stress score. The results are shown in Supplementary Table S1, and an increase in SBP and MAP was observed in the top quintile. However, ANOVA showed no significant difference in SBP, DBP, MAP, or the changes in BP.

## Discussion

From the results of this study, during the state of emergency, office BP significantly increased; however, home BP significantly decreased, and the ratio of white coat hypertension was significantly increased (*p* < 0.001). Decreased amounts of exercise and worsened diet compositions were observed in 39% and 17% of the patients, respectively; the patients with a total stress score ≥3 showed a significant increase not only in office systolic BP but also in home diastolic BP (*p* = 0.04 and 0.01, respectively), with a significant increase in the awareness of worsening stressors, except medication compliance (*p* < 0.001).

To prevent the spread of COVID-19, the first state of emergency announcement was issued on 7 April 2020. This announcement forced people to modify their activities, and many people felt increased physical and financial stress in their daily life. At that time, effective treatments and diagnostics for COVID-19 were in the very early stages of development. Polymerase chain reaction tests for the diagnosis of COVID-19 infection were difficult for general practitioners to carry out because of the risk of infection. This results in limitations in clinical practice, especially during the early stages of the pandemic. The Sagamihara Physicians Association planned to evaluate the effect of stress on BP management and how this effect was influenced by the COVID-19 state of emergency. Because SARS-CoV-2 enters human pulmonary cells by using angiotensin converting enzyme 2 (ACE2) as a receptor, there is serious concern about the adverse effects of hypertension as a condition contributing to the severity and poor prognosis of COVID-19. In fact, a high mortality rate (6%) has been reported in patients with hypertension in China [13], and nationwide Japanese media coverage has addressed this concern with great interest. As a result, many Japanese citizens were worried that COVID-19 may be more severe in patients with hypertension. This concern was confirmed by our present survey for stress. According to a February 2020 report on COVID-19 in Wuhan, there was no significant difference in the risk of intensive care unit admission in patients with or without hypertension [14]. Zhang et al. reported a significant decrease in mortality in patients treated with ACE inhibitors or angiotensin receptor agonizts [15]. In Japan, Matsuzawa reported that there was no significant relationship between the severity of COVID-19 and the use of renin-angiotensin system inhibitors; rather, these medications significantly decreased the complications of impaired consciousness [16].

The increase in BP in primary hypertensive patients associated with the COVID-19 pandemic in Turkey has already been reported. [17] This study, which involved Japanese outpatient patients, revealed a significant increase in office BP measurements: systolic BP increased by 2.1 mmHg and diastolic BP increased by 0.8 mmHg following the state of emergency announcement. In contrast, a significant decrease in home BP measurements (1.3 mmHg in systolic BP and 0.6 mmHg in diastolic BP) was observed during the state of emergency, and the rate of white coat hypertension increased during this period. It is well known that many world disasters can cause elevated BP; however, the influence of the COVID-19 state of emergency announcement on BP seemed different than the changes in BP caused by other disasters, such as earthquakes or typhoons, in Japan. Systolic and diastolic BP increased by 18 mmHg and 8 mmHg, respectively, in elderly patients following the Hanshin–Awaji earthquake in 1995 [18]. Additionally, systolic and diastolic BP increased by 4 mmHg and 3 mmHg, respectively, 1–3 weeks after the Great East Japan earthquake in 2011, and BP levels did not return to baseline in patients with hypertension or advanced stage chronic kidney disease until 5–7 weeks following the earthquake [19].

Because COVID-19 is an infectious disease, the risk of going outside and coming into contact with other people was highlighted during the state of emergency. During this time, patients who had to leave their homes to visit clinics may have become nervous, resulting in elevated office BP measurements, especially in patients experiencing chronic stress. White coat hypertension, rather than masked or continuous hypertension, presents a relatively high risk for cardiovascular disease compared to patients with well-controlled BP [20]. Several kinds of stress have been shown to increase the incidence of cardiovascular outcomes; for example, episodes of intense anger increased the incidence of acute myocardial infarction (AMI) with a relative risk of 2.3 (95% CI, 1.7–3.2) [21]. Additionally, the 1991 Persian Gulf War increased the incidence of AMI or sudden death by 92% [22], and the World Trade Center Attack on September 11, 2001, increased the incidence of tachyarrhythmia by 2.3% (95% CI, 1.1–4.9) among patients fitted with an implantable cardioverter defibrillator [23]. Future studies are needed to clarify whether white coat hypertension caused by issued state of emergency announcements may increase the incidence of cardiovascular disease.

Another reason for increased BP under the COVID-19 state of emergency is that some patients have refrained from visiting general practitioners. Amazing news was announced by the Japanese Ministry of Health, Labor, and Welfare. The total national medical expense from April 2020 to February 2021 was 38,300 billion yen, which was −4.4% compared to the same months of the previous year (https://www.mhlw.go.jp/topics/medias/month/21/02.html). One of the most likely reasons for this trend was a decrease in visits to clinics or hospitals for elective procedures due to the COVID-19 pandemic.

If patients with hypertension do not receive the necessary medical consultation (e.g., clinic visits), necessary antihypertensive therapy may be interrupted, leading to worsened BP management. Some patients exhibited well-controlled home BP and refrained from visiting general practitioners; however, our present study showed that 102 of 160 patients (64%) did not achieve the target office BP levels, even with well-controlled home BP. The number of patients with AMI in the hospital increased threefold in the three years following the Hurricane Katrina disaster in New Orleans in 2005 compared to before the disaster, and the increases in unemployment, noninsurance, refrain from visiting hospitals, and smoking were related to an increase in cardiovascular outcomes [24]. Therefore, it is necessary for general practitioners to allow patients to continue to visit clinics, even during emergencies. One strategy for encouraging patients to refrain from visiting hospitals is providing online medical care or continued prescription of the same medications using faxes. In fact, since the COVID-19 pandemic began in March 2020, these alternative measures have been strongly recommended by the Japanese government. However, future research and discussion will be needed to determine if these medical systems are as equally effective as traditional, in-person visits for BP control and the management of cardiovascular complications.

Celik et al. reported that anxiety disorders associated with the COVID-19 pandemic caused the deterioration of BP control in primary hypertensive patients using the Hospital Anxiety and Depression Scale (HADS) questionnaire. [17] In this study, we analyzed the total scores of these nine factors to assess stress and related changes in BP. Our study indicated a significant relationship between the total stress score and an increase in BP. However, we treated the nine factors equally, and a more in-depth scoring analysis could expand upon these findings. Because the Japanese government requested that citizens remain home during the state of emergency, a decrease in exercise and dietary adherence was predicted. In fact, the proportion of patients who reported a decrease in exercise was the largest compared to the other factors (39%), and the second largest factor was an increase in consumed calories (17%). Unlike acute stress, which mainly activates the autonomic nervous system, chronic stress lasting for several months activates the hypothalamus-pituitary-adrenal cortex system [25], which causes an increase in appetite. Our multiple linear regression analysis identified the increase in dietary intake as an important factor for the change in office MAP. Thus, general practitioners should give appropriate advice to patients regarding their dietary requirements during a state of emergency.

Requests for shortened business hours in the restaurant industry were pronounced during the state of emergency. Often, what patients eat for dinner or lunch outside of the home is a serious concern, especially for salt intake. Patients with hypertension are recommended to restrict their salt intake even when they cook at home, and they are advised to pay attention to the amount of salt contained in any processed food. In Japan, the salt content reported on food nutrition labels was changed from “amount of sodium” to “amount of salt” in 2015, making it easier for the general public to understand the amount of salt in packaged foods and monitor salt intake. The Japanese Society of Hypertension described the activity of reducing salt intake, and introduced suitable foods and reported how to cook to achieve reductions in salt intake. Such campaigns may be useful for helping individuals manage their salt intake and BP.

## Study limitations

Because this was a retrospective observational study, clinical measurements were only available at one point during the state of emergency. Because the first state of emergency lasted for approximately two months and the second or third state of emergency announcements were not lifted until 2021, long-term observations and multiplex investigations are needed in the future. The impact of the state of emergency varies from person to person and depends on where they live. Narrowing target groups of patients by factors such as geographical area, occupation, and family structure may be useful for the analysis of the relationship between state of emergency announcements and clinical findings. Additionally, changes in BP are only surrogate health markers, and studies on definitive outcomes, such as AMI, death, or other critical findings, should be performed in the future. Because 95% of the patients in this study had hypertension and 57% of them had DM, it is difficult to apply the results in this study to the general public. Examining members of the general public without hypertension is necessary; however, how to collect BP data will be a problem.

## Conclusion

A significant increase in office BP with the white coat phenomenon was observed during the state of emergency, as well as an increase in the related stress. To prevent cardiovascular events, general practitioners should pay careful attention to BP management and may need other strategies for BP management that differ from those in usual daily clinical practice during the COVID-19 pandemic.

## Supplementary information

Supplementary Table S1

Supplementary figure 1

Supplementary figure 2

## Data Availability

Data are available from Sagamihara Physicians Association Data Access for investigators bound by confidentiality agreements. Contact details: Sagamihara Physicians Association Address: 6-1-1 Fujimi, chuou-ku, Sagamihara city, Kanagawa prefecture, Japan, email: k-taishi@xc4.so-net.ne.jp, corresponding to KK.
