# Supplementary material for: Influence of stress induced by the first announced state of emergency due to coronavirus disease 2019 on outpatient blood pressure management in Japan
Source: Hypertens Res. 2021 Dec 24;45(4):675–85. doi: 10.1038/s41440-021-00832-w (PMC8705072; doi:10.1038/s41440-021-00832-w)
Supplement: Supplementary file 1 — Supplementary Table S1 [file 41440_2021_832_MOESM1_ESM.docx]

Supplementary Table S1 The comparison of office BP and home BP depending on the quantile groups.

| a) office BP (total n=674) | |  |  |  |  |  |  |  |  |  |  |  |  |
| --- | --- | --- | --- | --- | --- | --- | --- | --- | --- | --- | --- | --- | --- |
|  |  |  | SBP | | |  | DBP | | |  | MAP | | |
|  | The total stress score | n | Jan–Mar in 2020 | During the state of emergency | The change in SBP |  | Jan–Mar in 2020 | During the state of emergency | The change in DBP |  | Jan–Mar in 2020 | During the state of emergency | The change in MAP |
| First quintile | ≤-1 | 212 | 138.5±18.3 | 138.6±19.6 | 0.2 [-2.1, 2.4] |  | 78.6±12.1 | 79.0±12.2 | 0.3 [-1.0, 1.7] |  | 98.6±12.0 | 98.9±13.0 | 0.3 [-1.2, 1.7] |
| Second quintile | 0 | 120 | 136.3±19.5 | 137.3±19.5 | 1.1 [-2.3, 4.4] |  | 78.8±12.7 | 79.2±11.2 | 0.5 [-1.7, 2.6] |  | 97.9±13.5 | 98.6±12.0 | 0.7 [-1.7, 3.0] |
| Third quintile | 1 | 99 | 136.9±16.7 | 139.0±17.5 | 2.2 [-1.2, 5.5] |  | 77.4±12.3 | 79.1±12.3 | 1.6 [-0.5, 3.7] |  | 97.2±12.1 | 99.0±12.2 | 1.8 [-0.4, 4.0] |
| Fourth quintile | 2-3 | 129 | 136.0±16.7 | 139.0±15.7 | 3.0 [0.6, 5.4] |  | 77.3±12.5 | 78.6±11.1 | 1.3 [-0.3, 3.0] |  | 96.8±12.1 | 98.7±10.6 | 1.9 [0.3, 3.4] |
| Fifth quintile | ≥4 | 114 | 136.2±15.9 | 141.8±20.5 | 5.6 [2.5, 8.8] |  | 80.2±10.6 | 81.6±13.9 | 1.5 [-0.5, 3.4] |  | 98.8±10.8 | 101.7±14.5 | 2.9 [0.7, 5.0] |
| p-value by ANOVA |  |  | 0.67 | 0.47 | 0.07 |  | 0.36 | 0.31 | 0.76 |  | 0.62 | 0.28 | 0.29 |
|  |  |  |  |  |  |  |  |  |  |  |  |  |  |
| a) home BP (total n=486) | |  |  |  |  |  |  |  |  |  |  |  |  |
|  |  |  | SBP | | |  | DBP | | |  | MAP | | |
|  | The total stress score | n | Jan–Mar in 2020 | During the state of emergency | The change in SBP |  | Jan–Mar in 2020 | During the state of emergency | The change in DBP |  | Jan–Mar in 2020 | During the state of emergency | The change in MAP |
| First quintile | ≤-1 | 142 | 127.7±10.0 | 126.0±10.1 | -1.7 [-2.9, -0.4] |  | 75.7±9.3 | 74.8±9.2 | -0.7 [-1.6, 0.1] |  | 93.1±8.2 | 91.9±8.2 | -1.0 [-2.0, -0.1] |
| Second quintile | 0 | 81 | 127.9±8.8 | 127.6±9.7 | -0.5 [-2.0, 0.9] |  | 76.2±8.5 | 75.71±8.1 | -0.2 [-1.3, 0.8] |  | 93.4±7.4 | 93.0±7.1 | -0.3 [-1.3, 0.6] |
| Third quintile | 1 | 77 | 128.6±11.7 | 126.2±10.4 | -2.1 [-4.0, -0.2] |  | 77.5±9.8 | 75.9±9.5 | -1.5 [-2.7, -0.3] |  | 94.5±9.5 | 92.7±8.7 | -1.7 [-3.0, -0.4] |
| Fourth quintile | 2-3 | 99 | 127.4±10.2 | 126.1±11.4 | -1.5 [-2.9, 0.0] |  | 74.7±8.6 | 74.9±10.1 | -0.2 [-1.3, 0.9] |  | 92.3±7.6 | 91.9±9.4 | -0.6 [-1.7, 0.5] |
| Fifth quintile | ≥4 | 87 | 129.2±11.3 | 127.9±9.8 | -0.9 [-2.5, 0.8] |  | 76.5±8.7 | 76.3±9.0 | 0.0 [-1.2, 1.2] |  | 94.1±8.0 | 93.5±8.1 | -0.3 [-1.4, 0.9] |
| p-value by ANOVA |  |  | 0.77 | 0.59 | 0.68 |  | 0.34 | 0.73 | 0.36 |  | 0.39 | 0.60 | 0.41 |

Data shown are mean ± standard deviation, or mean [lower value of 95% of confidence interval, upper value of 95% CI].

Abbreviations: ANOVA, analysis of variance; DBP, diastolic blood pressure; MAP, mean arterial pressure, SBP, systolic blood pressure.
