# Supplementary figures and images for: Influence of stress induced by the first announced state of emergency due to coronavirus disease 2019 on outpatient blood pressure management in Japan
Source: Hypertens Res. 2021 Dec 24;45(4):675–85. doi: 10.1038/s41440-021-00832-w (PMC8705072; doi:10.1038/s41440-021-00832-w)

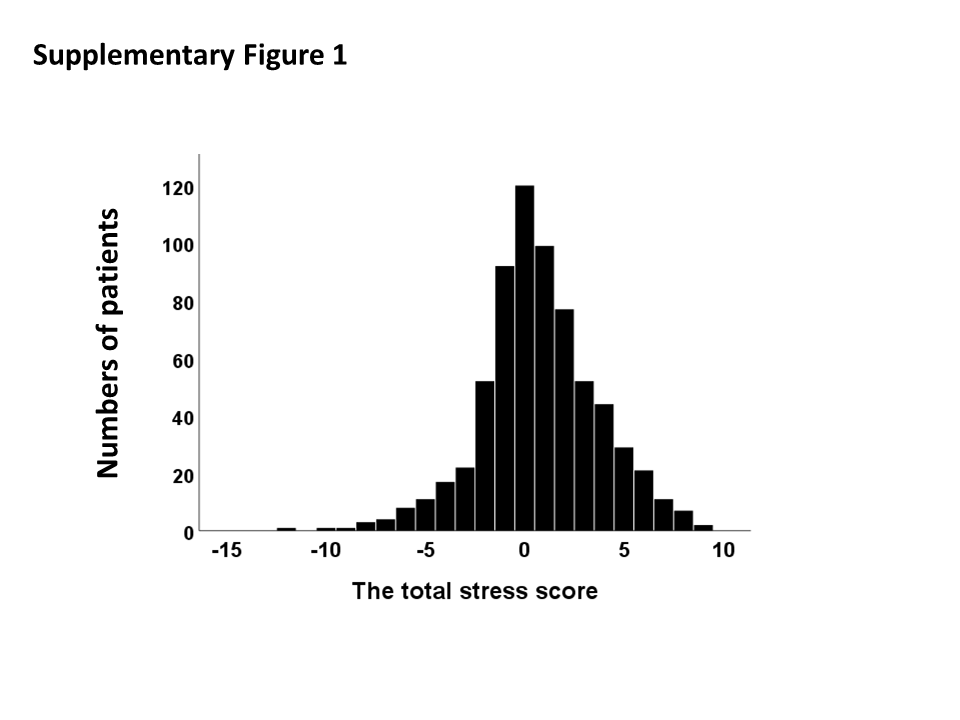

Supplement: Supplementary file 2 — Supplementary figure 1 [file 41440_2021_832_MOESM2_ESM.tif]

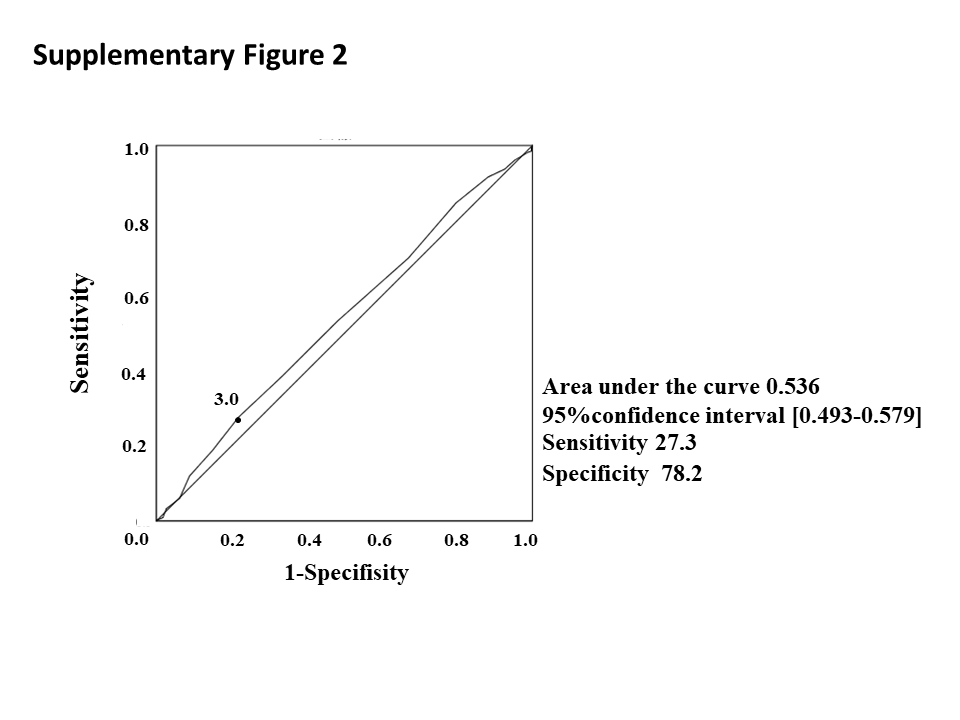

Supplement: Supplementary file 3 — Supplementary figure 2 [file 41440_2021_832_MOESM3_ESM.tif]
